# Relationship between Interpersonal Sensitivity Measure score and clinical symptoms in patients with major psychiatric disorders and healthy individuals

**DOI:** 10.1002/pcn5.18

**Published:** 2022-06-15

**Authors:** Shinsuke Hidese, Junko Matsuo, Ikki Ishida, Yuuki Yokota, Miho Ota, Kotaro Hattori, Hiroshi Kunugi

**Affiliations:** ^1^ Department of Mental Disorder Research, National Institute of Neuroscience National Center of Neurology and Psychiatry Kodaira Japan; ^2^ Department of Psychiatry Teikyo University School of Medicine Itabashi‐ku Japan; ^3^ Department of Psychiatry, National Center Hospital National Center of Neurology and Psychiatry Kodaira Japan; ^4^ Department of Behavioral Medicine, National Institute of Mental Health National Center of Neurology and Psychiatry Kodaira Japan; ^5^ Department of Neuropsychiatry, Division of Clinical Medicine, Faculty of Medicine University of Tsukuba Tsukuba Japan; ^6^ Medical Genome Center National Center of Neurology and Psychiatry Kodaira Japan

**Keywords:** healthy individuals, Interpersonal Sensitivity Measure, major psychiatric disorders, symptom

## Abstract

**Aim:**

We compared the Interpersonal Sensitivity Measure (IPSM) scores between diagnostic groups and examined the relationship between IPSM scores and clinical variables.

**Methods:**

This study included 166 patients with schizophrenia, 47 patients with bipolar disorder (BD) Ⅰ, 110 patients with BD Ⅱ, 380 patients with major depressive disorder (MDD), and 558 healthy individuals. Symptoms were assessed using the Positive and Negative Syndrome Scale (PANSS), Young Mania Rating Scale, 21‐item Hamilton Depression Rating Scale (HAMD‐21), and Pittsburgh Sleep Quality Index (PSQI).

**Results:**

The IPSM interpersonal awareness, separation anxiety, timidity, fragile inner self, and total scores were significantly higher in all the patient groups compared with healthy individuals (corrected *p* < 0.05). The IPSM need for approval score was significantly higher in patients with BD Ⅰ and those with BD Ⅱ than in those with schizophrenia or MDD (corrected *p* < 0.05). The PSQI total score and PANSS general psychopathology score, HAMD‐21 delusion subscale score, HAMD‐21 total score, and HAMD‐21 core subscale score and PSQI sleep disturbance subscale score were significantly and positively correlated with the IPSM total score in patients with schizophrenia, those with BD Ⅰ, those with BD Ⅱ, and those with MDD, respectively, while the PSQI total score and daytime dysfunction subscale score were significantly and positively correlated with the IPSM total score in healthy individuals (corrected *p* < 0.05).

**Conclusion:**

Our data suggest that higher interpersonal sensitivity may play a role in the development of major psychiatric disorders and may be involved in some clinical symptom formations.

## INTRODUCTION

The Interpersonal Sensitivity Measure (IPSM) was developed as a self‐report measure of interpersonal sensitivity (IPS).^1^ The IPSM consists with five subscales (i.e., interpersonal awareness, need for approval, separation anxiety, timidity, and fragile inner self), which correspond to sensitivity to interpersonal interactions, the wish to be liked and to make others happy, anxiety about interpersonal relations, lack of assertiveness, and aspects of self‐worth, respectively.^2^ The IPSM has been reported to be related to neuroticism,^1^ which has been suggested in the personality trait related with psychopathology and underlying mechanism in common psychiatric disorders.^3^, ^4^, ^5^ However, to our knowledge, the significance of the five IPSM constructs in relation to psychopathology of different psychiatric diagnostic groups has not been investigated in a single cohort.

IPS has been included in the psychopathology of patients with schizophrenia.^6^, ^7^ A study reported that compared with healthy controls, 62 patients with at‐risk mental state had higher IPSM interpersonal awareness, separation anxiety, fragile inner self, and total scores.^8^ Another study reported that 34 patients with first‐episode psychosis had higher IPSM interpersonal awareness, separation anxiety, and fragile inner self scores than healthy controls.^9^ These suggest that higher IPSM scores (i.e., interpersonal awareness, separation anxiety, and fragile inner self) are associated with schizophrenia psychopathology; however, studies on longer duration of schizophrenia or psychotic symptoms have still been scarce.

IPS assessed using the Temperament and Personality Questionnaire, which comprises 10 subscales that correspond to the five‐factor model and may assess personality traits associated with depression,^10^ was higher in 158 patients with bipolar disorder (BD) Ⅱ than in 258 patients with unipolar depression; however, no difference in IPS was noted between patients with BD Ⅰ (*n* = 27) and those with unipolar depression or BD Ⅱ.^11^ The IPSM total score was associated with cyclothymic‐sensitive affective temperament in 89 patients with BD Ⅰ.^12^ Considering these studies, characteristics of the IPSM scores may be different between BD Ⅰ and Ⅱ and possibly correlate with BD symptoms, while there has been no previous information on the five IPSM constructs examined in patients with BD or their comparison with those in healthy individuals.

Higher IPSM has been shown in patients with depression,^1^ especially in those with non‐melancholic depression compared with melancholic depression.^13^, ^14^, ^15^ Higher IPSM was associated with poorer outcome of depressive episode in 111 patients.^16^ IPSM interpersonal awareness, need for approval, separation anxiety, and total scores were higher in individuals with a history of major depressive disorder (MDD) than in 306 individuals without a history of MDD,^17^ indicating positive correlations of the IPSM need for approval, fragile inner self, and total scores with the Beck Depression Inventory score. Higher IPSM total score was associated with antidepressant treatment‐resistant depression in 199 patients with MDD,^18^ whereas lower IPSM total score was associated with “return to work” in 88 patients with MDD,^19^ indicating positive correlations between the IPSM total and 17‐item Hamilton Rating Scale for Depression scores. These suggest that higher IPSM scores and their sum (i.e., total score) are associated with depressive symptoms and poorer prognosis in MDD.

The association of the IPSM interpersonal awareness, separation anxiety, timidity, and fragile inner self scores with core beliefs of negative‐self was assessed using the Brief Core Schema Scales in 335 healthy individuals^20^ and found that the IPSM interpersonal awareness and separation anxiety scores were negatively associated with male sex. In the Brief Symptom Inventory, IPS was positively associated with suicidal ideation in 312 community‐based participants with no psychiatric disorders.^21^ These findings prompted us to investigate possible association between the IPSM scores and clinical variables in healthy individuals.

Although previous studies have dealt with IPSM scores in patients with schizophrenia,^8^, ^9^ those with BD,^11^, ^12^ those with MDD,^16^, ^17^, ^18^, ^19^ and healthy individuals,^20^, ^21^ the relationship between the IPSM scores and general clinical variables, such as symptom scores, have not been investigated, except in studies by Sakado et al.^17^ and Ogawa et al.^19^ Furthermore, patients with a relatively long duration of schizophrenia were not included. Based on previous reviews regarding the association between IPS and psychopathology of schizophrenia^6^, ^7^ and depressive disorders (i.e., BD and MDD),^1^, ^5^ this study aimed to compare the IPSM scores between the diagnostic groups and examine the clinical variables related to IPSM scores in a single cohort comprised with a relatively large number of patients with major psychiatric disorders and healthy individuals. This study could not be based on specific theory relating IPS to some clinical significance in a certain group of patients; however, considering that IPS is a personality trait that could be a vulnerability factor for the development of psychiatric disorders^1^, ^2^, ^3^, ^4^, ^5^ and may predict symptoms, we hypothesized that the IPSM scores would be higher in major psychiatric disorders (i.e., schizophrenia, BD, and MDD), and be risk factors for symptom or sleep state in the patients and healthy individuals.

## METHODS

### Participants

We investigated 166 patients with schizophrenia (mean ± standard deviation age: 38.1 ± 11.6 years; 84 men and 82 women), 47 patients with BD Ⅰ (mean ± standard deviation age: 43.2 ± 11.3 years; 23 men and 24 women), 110 patients with BD Ⅱ (mean ± standard deviation age: 40.0 ± 10.4 years; 53 men and 57 women), 380 patients with MDD (mean ± standard deviation age: 40.9 ± 11.9 years; 180 men and 200 women), and 558 healthy individuals (mean ± standard deviation age: 45.5 ± 14.5 years; 217 men and 341 women). Both depressed and manic state were included in patients with BD, while both remitted and unremitted state were included in patients with BD and those with MDD. All participants (both patients and healthy individuals) were enrolled through recruitment forms distributed to fixed locations inside the National Center of Neurology and Psychiatry (NCNP) hospital and through the community outside the NCNP (i.e., advertisements in a free local magazine and announcements on our website) and contacted the researchers by telephone. All participants were screened for axis I psychiatric disorders using the Japanese version of the Mini‐International Neuropsychiatric Interview (MINI)^22^, ^23^ by trained psychiatrists. Patients were diagnosed according to the *Diagnostic and Statistical Manual of Mental Disorders* fourth edition criteria^24^ based on the results of the MINI and medical records, if available. None of the healthy individuals had axis I psychiatric disorders or had received psychiatric services. Patients and healthy individuals with a medical history of neurological diseases, severe head injury, substance abuse, or intellectual disability were excluded. All participants signed an informed consent form after they were explained the purpose and protocol of the study. The study protocol was approved by the ethics committee of the NCNP and complied with the Declaration of Helsinki.^25^

### Clinical and psychological assessments

Based on a previous report by Boyce and Parker,^1^ IPS was assessed using the Japanese version of the IPSM whose reliability and validity have been confirmed,^26^ which contains a total of 36 items based on a four‐point Likert scale and the sum was used as the scores of the five components (i.e., interpersonal awareness, need for approval, separation anxiety, timidity, and fragile inner self).^17^ The symptom severity of patients was assessed using the Japanese version of the Positive and Negative Syndrome Scale (PANSS),^27^, ^28^ the Young Mania Rating Scale (YMRS),^29^ and the GRID 21‐item Hamilton Depression Rating Scale (HAMD‐21)^30^, ^31^ to evaluate psychotic, manic, and depressive symptoms, respectively. The HAMD‐21 has six subscales, namely core, sleep, activity, psychic anxiety, somatic anxiety, and delusion, as described by Seretti et al.^32^ The sleep state was assessed using the Japanese version of the Pittsburgh Sleep Quality Index (PSQI).^33^ Patients' daily doses of antipsychotics and antidepressants were converted to chlorpromazine‐equivalent and imipramine‐equivalent doses, respectively, as described in a previous Japanese study.^34^

### Statistical analyses

Continuous, ordinal, and dichotomous variables were compared between the patient and healthy individual groups using the Student's or Welch's *t*‐test, Kolmogorov–Smirnov *Z* test, and *χ* ^2^ test, respectively. The IPSM scores were compared between the diagnostic groups using the one‐way multivariate analysis of covariance (MANCOVA), after controlling for age and sex, and Bonferroni corrections were applied to account for each two‐group comparison among the five diagnostic groups. Multiple regression analyses were performed for the IPSM scores (objective variables) with all the clinical variables in each diagnostic group as explanatory variables using the stepwise method. The correlations of the continuous and ordinal (i.e., PSQI and HAMD‐21 subscales) variables with the IPSM scores were determined using Pearson's and Kendall's rank correlation coefficients, respectively. The IPSM scores were compared by dichotomous variables (i.e., sex and psychotropic medication use) in each diagnostic group using the Student's or Welch's *t*‐test. The IPSM scores were compared between the lifetime suicide attempters and non‐attempters in each diagnostic group according to the number of attempts using the MANCOVA after controlling for age, sex, and psychotropic medication use. MANCOVAs were conducted always incorporating age and sex into covariates to preclude their nuisance effects on the IPSM scores. Bonferroni corrections were applied to account for multiple testing of the six IPSM scores using the MANCOVA, multiple regression analyses, correlational analyses, and Student's or Welch's *t*‐test. In the analyses of the six IPSM scores, a *p*‐value of <0.0083 (<0.05/6) was considered significant. The effect sizes were calculated using *r* for the unpaired *t*‐tests, *D* for the Kolmogorov–Smirnov *Z* tests, *φ* for the *χ* ^2^ tests, and *η* ^2^ for the MANCOVA. Goodness of fit for the multiple regression analysis was evaluated using the adjusted *R* ^2^. Statistical analysis was performed using the Statistical Package for the Social Sciences (Version 27.0; IBM Japan Ltd). All statistical tests were two‐tailed, and *p*‐values <0.05 were considered significant.

## RESULTS

The clinical characteristics of the participants are presented in Table 1. Compared with healthy individuals, all patients had significantly higher body mass index and patients with BD Ⅱ had significantly higher and patients with schizophrenia had significantly lower education level (nominal *p* < 0.05). The PSQI total score was significantly higher in all the patient groups and the number of lifetime suicide attempts was significantly higher in patients with schizophrenia, those with BD Ⅱ, and those with MDD than in healthy individuals (nominal *p* < 0.001).

**Table 1:** The clinical characteristics of the participants

	Schizophrenia (*n* = 166)		Bipolar disorderⅠ (*n* = 47)		Bipolar disorderⅡ (*n* = 110)		Major depressive disorder (*n* = 380)		Healthy individual (*n* = 558)
	Mean ± Standard deviation	vs. control	Mean ± Standard deviation	vs. control	Mean ± Standard deviation	vs. control	Mean ± Standard deviation	vs. control	Mean ± Standard deviation
Age (years)	38.1 ± 11.6	Welch's *t* (331.0) = 6.77, ***p*** = **5.9.E−11**, *r* = 0.349	43.2 ± 11.3	Welch's *t* (59.5) = 1.32, *p* = 0.19, *r* = 0.169	40.0 ± 10.4	Welch's *t* (202.2) = 4.73, ***p*** = **4.3.E−6**, *r* = 0.316	40.9 ± 11.9	Welch's *t* (904.5) = 5.29, ***p*** = **1.6.E−7**, *r* = 0.174	45.5 ± 14.5
Sex, men (%)	84 (50.6)	*χ* ^2^ (1) = 7.23, ***p*** = **0.007**, *φ* = −0.100	23 (48.9)	*χ* ^2^ (1) = 1.83, *p* = 0.18, *φ* = −0.055	53 (48.2)	*χ* ^2^ (1) = 3.30, *p* = 0.07, *φ* = −0.070	180 (47.4)	*χ* ^2^ (1) = 6.06, ***p*** = **0.010**, *φ* = −0.084	217 (38.9)
Body mass index (kg/m^2^)	23.6 ± 4.3	Welch's *t* (204.2) = −4.17, ***p*** = **4.6.E−6**, *r* = 0.281	24.0 ± 4.6	Welch's *t* (47.6) = −2.78, ***p*** = **0.008**, *r* = 0.374	23.5 ± 4.7	Welch's *t* (125.2) = −3.06, ***p*** = **0.003**, *r* = 0.264	22.7 ± 4.1	Welch's *t* (659.1) = −2.25, ***p*** = **0.025**, *r* = 0.088	22.1 ± 3.2
Education (years)	13.9 ± 2.4	Student's *t* (721) = 4.59, ***p*** = **5.0.E−6**, *r* = 0.169	14.8 ± 3.1	Student's *t* (602) = 0.04, *p* = 0.97, *r* = 0.002	15.4 ± 2.3	Student's *t* (665) = −2.36, ***p*** = **0.018**, *r* = 0.095	14.8 ± 2.2	Student's *t* (934) = −0.17, *p* = 0.87, *r* = 0.006	14.8 ± 2.2
Pittsburgh Sleep Quality Index	7.5 ± 3.5	Welch's *t* (206.8) = −8.06, ***p*** = **6.2.E−14**, *r* = 0.489	9.0 ± 4.0	Welch's *t* (47.1) = −6.35, ***p*** = **7.9.E−8**, *r* = 0.680	9.3 ± 3.4	Welch's *t* (124.9) = −11.95, ***p*** = **2.0.E−22**, *r* = 0.731	8.3 ± 3.6	Welch's *t* (596.6) = −14.53, ***p*** = **3.8.E−14**, *r* = 0.512	5.1 ± 2.6
Sleep quality	1.3 ± 0.8	*Z* = 1.56, ***p*** = **0.016**, *D* = 0.142	1.5 ± 0.9	*Z* = 1.30, *p* = 0.07, *D* = 0.202	1.6 ± 0.8	*Z* = 2.56, ***p*** = **3.9.E‐6**, *D* = 0.276	1.6 ± 0.8	*Z* = 3.67, ***p*** **≒ 0**, *D* = 0.250	1.1 ± 0.7
Sleep latency	1.4 ± 1.1	*Z* = 2.75, ***p*** = **5.5.E−7**, *D* = 0.250	1.5 ± 1.1	*Z* = 2.24, ***p*** = **9.0.E−5**, *D* = 0.347	1.7 ± 1.1	*Z* = 3.54, ***p*** **≒ 0**, *D* = 0.382	1.4 ± 1.1	*Z* = 3.76, ***p*** **≒ 0**, *D* = 0.256	0.8 ± 0.9
Sleep duration	0.8 ± 1.0	*Z* = 4.27, ***p*** **≒ 0**, *D* = 0.388	1.3 ± 1.0	*Z* = 0.54, *p* = 0.93, *D* = 0.084	1.1 ± 1.1	*Z* = 2.21, ***p*** = **1.1.E−4**, *D* = 0.238	1.1 ± 1.0	*Z* = 2.50, ***p*** = **7.0.E−6**, *D* = 0.170	1.4 ± 0.9
Habitual sleep efficiency	0.4 ± 0.8	*Z* = 1.22, *p* = 0.10, *D* = 0.111	0.5 ± 0.9	*Z* = 1.05, *p* = 0.22, *D* = 0.163	0.6 ± 0.9	*Z* = 1.74, ***p*** = **4.7.E−3**, *D* = 0.188	0.4 ± 0.8	*Z* = 1.32, *p* = 0.063, *D* = 0.090	0.2 ± 0.6
Sleep disturbance	1.0 ± 0.6	*Z* = 1.52, ***p*** = **0.020**, *D* = 0.138	1.0 ± 0.6	*Z* = 0.90, *p* = 0.40, *D* = 0.140	1.1 ± 0.6	*Z* = 1.80, ***p*** = **3.1.E−3**, *D* = 0.194	1.0 ± 0.6	*Z* = 2.04, ***p*** = **4.7.E−4**, *D* = 0.139	0.8 ± 0.5
Use of sleeping medication	1.4 ± 1.4	*Z* = 5.54, ***p*** **≒ 0**, *D* = 0.503	1.5 ± 1.4	*Z* = 3.46, ***p*** **≒ 0**, *D* = 0.537	1.7 ± 1.4	*Z* = 5.52, ***p*** **≒ 0**, *D* = 0.595	1.4 ± 1.4	*Z* = 6.83, ***p*** **≒ 0**, *D* = 0.465	0.1 ± 0.5
Daytime dysfunction	1.2 ± 0.9	*Z* = 2.35, ***p*** = **3.1.E−5**, *D* = 0.213	1.5 ± 1.0	*Z* = 2.84, ***p*** = **2.0.E−7**, *D* = 0.440	1.5 ± 0.7	*Z* = 3.57, ***p*** **≒ 0**, *D* = 0.385	1.4 ± 0.8	*Z* = 4.80, ***p*** ** ≒ 0**, *D* = 0.327	0.7 ± 0.7
Number of lifetime suicide attempts	0.5 ± 1.3	*Z* = 2.84, ***p*** = **1.9.E−7**, *D* = 0.251	0.2 ± 0.4	*Z* = 0.97, *p* = 0.31, *D* = 0.148	0.8 ± 2.3	*Z* = 2.60, ***p*** = **2.8.E−6**, *D* = 0.271	0.4 ± 1.5	*Z* = 2.03, ***p*** = **5.3.E−4**, *D* = 0.135	0.0 ± 0.0
Age of onset (years)	23.8 ± 8.5		28.1 ± 10.6		28.2 ± 10.6		31.2 ± 12.2		
Duration of illness (years)	14.1 ± 9.5		13.6 ± 8.9		11.2 ± 8.4		6.7 ± 6.3		
Chlorpromazine‐equivalent dose (mg/day)									
Total	501.0 ± 739.8		160.1 ± 352.0		87.3 ± 189.7		36.8 ± 110.7		
Typical	95.1 ± 233.1		5.2 ± 18.4		4.3 ± 15.8		9.3 ± 59.8		
Atypical	406.5 ± 726.7		154.9 ± 353.5		83.0 ± 189.4		27.6 ± 92.8		
Imipramine‐equivalent dose (mg/day)	25.4 ± 86.0		17.8 ± 72.4		81.0 ± 135.5		84.4 ± 134.2		
Psychotropic medication use, *n* (%)	139 (83.7)		23 (48.9)		67 (60.9)		192 (50.2)		
PANSS total	59.1 ± 16.2								
PANSS positive	13.9 ± 4.7								
PANSS negative	15.1 ± 5.8								
PANSS general psychopathology	30.1 ± 8.2								
Young Mania Rating Scale			4.2 ± 6.6		2.4 ± 3.6				
21‐item Hamilton Depression Rating Scale			9.6 ± 7.9		12.3 ± 8.1		11.8 ± 7.9		
Core			4.7 ± 4.2		5.9 ± 4.1		6.3 ± 4.4		
Sleep			1.5 ± 1.7		1.7 ± 1.7		1.6 ± 1.6		
Activity			1.4 ± 1.6		1.8 ± 1.5		1.9 ± 1.7		
Psychic anxiety			1.0 ± 1.3		1.2 ± 1.1		1.3 ± 1.3		
Somatic anxiety			1.6 ± 1.8		1.9 ± 1.6		2.0 ± 1.7		
Delusion			1.3 ± 1.6		1.6 ± 1.8		1.4 ± 1.5		

Abbreviation: PANSS, Positive and Negative Syndrome Scale.

Nominal significant *p*‐values (*p* < 0.05) are shown in bold cases.

**Figure 1:**
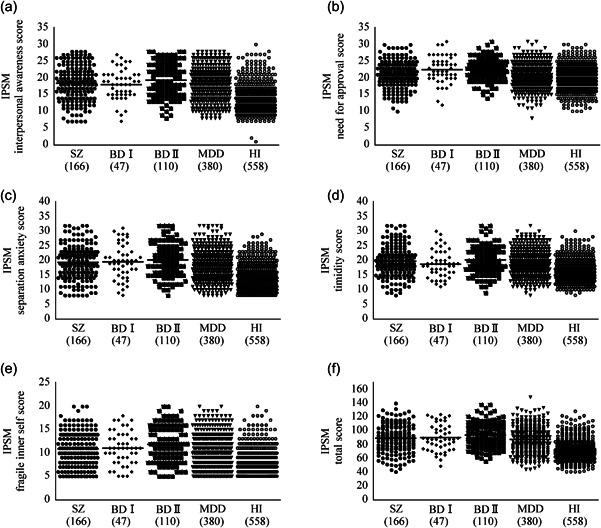
Dot plots of the Interpersonal Sensitivity Measure (IPSM) scores in the diagnostic groups. The (a) interpersonal awareness, (b) need for approval, (c) separation anxiety, (d) timidity, (e) fragile inner self, and (f) total scores in patients with schizophrenia (SZ), those with bipolar disorder (BD) Ⅰ, those with BD Ⅱ, those with major depressive disorder (MDD), and healthy individuals (HI). Horizontal lines in dot plots represent mean values. Numbers in parentheses represent the number of the participants in the diagnostic groups.

Comparisons of the IPSM scores between the diagnostic groups are presented in Table 2. The interpersonal awareness, separation anxiety, timidity, fragile inner self, and total scores were significantly higher in all the patient groups than in healthy individuals (corrected *p* < 0.05). No significant difference was noted between patients with schizophrenia or MDD and healthy individuals for the need for approval score. However, the need for approval score was significantly higher in patients with BD Ⅱ and those with BD Ⅰ than in patients with schizophrenia and those with MDD, respectively (corrected *p* < 0.05). Furthermore, the need for approval, fragile inner self, and total scores were significantly higher in patients with BD Ⅱ than in those with MDD (corrected *p* < 0.05). No significant difference was noted in all IPSM scores between patients with BD Ⅰ and those with BD Ⅱ. Dot plots of the IPSM scores in the diagnostic groups are shown in Figure 1.

**Table 2:** Comparisons of the Interpersonal Sensitivity Measure scores between the diagnostic groups

	Schizophrenia	BD Ⅰ	BD Ⅱ	MDD	Healthy individual	
	Mean ± Standard deviation	Mean ± Standard deviation	Mean ± Standard deviation	Mean ± Standard deviation	Mean ± Standard deviation	Statistical comparison
1. Interpersonal awareness	18.5 ± 5.5	18.1 ± 4.5	19.4 ± 5.1	18.5 ± 5.6	13.9 ± 4.3	*p* = 1.5.E**−**17 (schizophrenia vs. control), *p* = 2.7.E**−**7 (BD Ⅰ vs. control), *p* = 8.4.E**−**22 (BD Ⅱ vs. control), *p* = 4.3.E**−**35 (MDD vs. control)
2. Need for approval	21.0 ± 4.2	22.6 ± 4.1	22.5 ± 3.4	20.7 ± 3.8	19.9 ± 3.7	*p* = 1.6.E**−**5 (BD Ⅰ vs. control), *p* = 4.9.E**−**9 (BD Ⅱ vs. control), *p* = 4.1.E**−**3 (BD Ⅱ vs. schizophrenia), *p* = 3.0.E**−**3 (BD Ⅱ vs. MDD), *p* = 7.8.E**−**5 (BD Ⅱ vs. MDD)
3. Separation anxiety	19.3 ± 6.0	19.7 ± 5.9	20.3 ± 5.7	18.5 ± 5.4	14.2 ± 4.4	*p* = 8.8.E**−**22 (schizophrenia vs. control), *p* = 2.2.E**−**11 (BD Ⅰ vs. control), *p* = 3.1.E**−**25 (BD Ⅱ vs. control), *p* = 8.4.E**−**30 (MDD vs. control)
4. Timidity	19.7 ± 5.4	18.8 ± 4.6	20.1 ± 4.9	19.4 ± 4.8	16.4 ± 3.7	*p* = 3.4.E**−**12 (schizophrenia vs. control), *p* = 6.7.E**−**3 (BD Ⅰ vs. control), *p* = 1.7.E**−**12 (BD Ⅱ vs. control), *p* = 1.4.E**−**18 (MDD vs. control)
5. Fragile inner self	10.9 ± 3.8	11.1 ± 3.5	11.9 ± 3.9	10.4 ± 3.8	7.7 ± 2.9	*p* = 6.4.E**−**18 (schizophrenia vs. control), *p* = 1.6.E**−**9 (BD Ⅰ vs. control), *p* = 1.3.E**−**25 (BD Ⅱ vs. control), *p* = 2.8.E**−**25 (MDD vs. control), *p* = 1.2.E**−**3 (BD Ⅱ vs. MDD)
6. Total	89.6 ± 20.7	90.2 ± 19.2	94.3 ± 18.7	87.4 ± 18.8	72.1 ± 15.6	*p* = 2.4.E**−**20 (schizophrenia vs. control), *p* = 1.5.E**−**10 (BD Ⅰ vs. control), *p* = 1.4.E**−**27 (BD Ⅱ vs. control), *p* = 1.1.E**−**30 (MDD vs. control), *p* = 4.1.E**−**3 (BD Ⅱ vs. MDD)

Abbreviations: BD, bipolar disorder; MDD, major depressive disorder.

Only corrected significant *p*‐values (*p* < 0.0083) are shown in exponents.

Multiple regression analyses for the IPSM scores in each diagnostic group are shown in Tables 3, 4, 5, 6, 7. In patients with schizophrenia, the PSQI total score and PANSS general psychopathology score were significantly and positively correlated, whereas age was significantly and negatively correlated, with the IPSM total score (corrected *p* < 0.05; Table 3). In patients with BD Ⅰ, the HAMD‐21 delusion subscale score was significantly and positively correlated with the IPSM total score (corrected *p* < 0.05; Table 4). In patients with BD Ⅱ, the HAMD‐21 total score was significant and positively correlated with the IPSM total score (corrected *p* < 0.05; Table 5). In patients with MDD, the HAMD‐21 core subscale score, duration of illness, and PSQI sleep disturbance subscale score were significant and positively correlated, whereas age and HAMD‐21 activity subscale scores were significantly and negatively correlated with the IPSM total score (corrected *p* < 0.05; Table 6). In healthy individuals, the PSQI total score and PSQI daytime dysfunction subscale score were significantly and positively correlated, whereas age was significantly and negatively correlated, with the IPSM total score (corrected *p* < 0.05; Table 7).

**Table 3:** Multiple regression analyses for the Interpersonal Sensitivity Measure scores in patients with schizophrenia

1. Interpersonal awareness			2. Need for approval		
Adjusted *R* ^2^ = 0.266	*β*	*p*	Adjusted *R* ^2^ = 0.077	*β*	*p*
(Constant)		**2.4.E−10**	(Constant)		**3.0.E−33**
PSQI total	0.33	**2.5.E−05**	Age of onset	−0.21	0.010
Age	−0.25	**1.1.E−03**	PANSS negative	−0.22	0.009
PANSS general psychopathology	0.38	**5.3.E−04**	PSQI total	0.19	0.026
PANSS negative	−0.22	0.036			

3. Separation anxiety			4. Timidity		
Adjusted *R* ^2^ = 0.299	*β*	*p*	Adjusted *R* ^2^ = 0.167	*β*	*p*
(Constant)		**1.0.E−09**	(Constant)		**1.0.E−24**
PSQI total	0.33	**2.0.E−05**	PSQI total	0.35	**1.4.E−05**
PANSS general psychopathology	0.48	**1.1.E−05**	Age of onset	−0.23	**3.8.E−03**
Age of onset	−0.25	**7.4.E−04**	Chlorpromazine‐equivalent dose typical	−0.16	0.037
PANSS negative	−0.32	**2.3.E−03**			

5. Fragile inner self			6. Total		
Adjusted *R* ^2^ = 0.229	*β*	*p*	Adjusted *R* ^2^ = 0.260	*β*	*p*
(Constant)		**1.5.E−08**	(Constant)		**7.3.E−17**
PANSS general psychopathology	0.24	**2.3.E−03**	PSQI total	0.34	**1.5.E−05**
Age	−0.29	**2.0.E−04**	Age	−0.25	**8.1.E−04**
PSQI total	0.25	**1.5.E−03**	PANSS general psychopathology	0.36	**1.1.E−03**
			PANSS negative	−0.24	0.023

Abbreviations: PANSS, Positive and Negative Syndrome Scale; PSQI, Pittsburgh Sleep Quality Index.

Corrected significant *p*‐values are shown in bold exponents (*p* < 0.0083).

**Table 4:** Multiple regression analyses for the Interpersonal Sensitivity Measure scores in patients with bipolar disorder Ⅰ

1. Interpersonal awareness			2. Need for approval		
Adjusted *R* ^2^ = 0.240	*β*	*p*	Adjusted *R* ^2^ = 0.199	*β*	*p*
(Constant)		**5.3.E−20**	(Constant)		**2.2.E−09**
HAMD‐21 delusion	0.51	**1.6.E−03**	Psychotropic medication use	0.41	0.013
			Sex	0.32	0.044

3. Separation anxiety			4. Timidity		
Adjusted *R* ^2^ = 0.532	*β*	*p*	Adjusted *R* ^2^ = 0.145	*β*	*p*
(Constant)		**6.7.E−03**	(Constant)		**3.7.E−19**
HAMD‐21 delusion	0.66	**4.1.E−06**	HAMD‐21 delusion	0.41	0.014
Number of lifetime suicide attempts	−0.32	0.012			
Body mass index	0.26	0.039			

5. Fragile inner self			6. Total		
Adjusted *R* ^2^ = 0.308	*β*	*p*	Adjusted *R* ^2^ = 0.281	*β*	*p*
(Constant)		**1.5.E−13**	(Constant)		**1.1.E−22**
Young Mania Rating Scale	−0.37	0.015	HAMD‐21 delusion	0.55	**6.2.E−04**
Number of lifetime suicide attempts	−0.35	0.021			
PSQI sleep latency	0.31	0.044			

*Note*: Sex was coded as male: 1 and female: 2.

Abbreviations: HAMD‐21, 21‐item Hamilton Depression Rating Scale; PSQI, Pittsburgh Sleep Quality Index.

Corrected significant *p*‐values are shown in bold exponents (*p* < 0.0083).

**Table 5:** Multiple regression analyses for the Interpersonal Sensitivity Measure scores in patients with bipolar disorder Ⅱ

1. Interpersonal awareness			2. Need for approval		
Adjusted *R* ^2^ = 0.277	*β*	*p*	Adjusted *R* ^2^ = 0.040	*β*	*p*
(Constant)		**3.6.E−15**	(Constant)		**3.9.E−64**
HAMD‐21 delusion	0.31	**2.4.E−03**	Imipramine‐equivalent dose	−0.23	0.038
HAMD‐21 sleep	0.36	**6.1.E−04**			
PSQI sleep latency	−0.25	0.014			
Sex	0.20	0.043			

3. Separation anxiety			4. Timidity		
Adjusted *R* ^2^ = 0.188	*β*	*p*	Adjusted *R* ^2^ = 0.119	*β*	*p*
(Constant)		**1.7.E−29**	(Constant)		**9.6.E−18**
HAMD‐21 total	0.43	**4.5.E−05**	PSQI sleep disturbance	0.29	**6.4.E−03**
Imipramine‐equivalent dose	−0.22	0.032	Age of onset	−0.25	0.020

5. Fragile inner self			6. Total		
Adjusted *R* ^2^ = 0.121	*β*	*p*	Adjusted *R* ^2^ = 0.136	*β*	*p*
(Constant)		**6.6.E−34**	(Constant)		**4.9.E−40**
HAMD‐21 delusion	0.36	**7.4.E−04**	HAMD‐21 total	0.37	**6.7.E−04**
			Imipramine‐equivalent dose	−0.21	0.042

*Note*: Sex was coded as male: 1 and female: 2.

Abbreviations: HAMD‐21, 21‐item Hamilton Depression Rating Scale; PSQI, Pittsburgh Sleep Quality Index.

Corrected significant *p*‐values are shown in bold exponents (*p* < 0.0083).

**Table 6:** Multiple regression analyses for the Interpersonal Sensitivity Measure scores in patients with major depressive disorder

1. Interpersonal awareness			2. Need for approval		
Adjusted *R* ^2^ = 0.195	*β*	*p*	Adjusted *R* ^2^ = 0.055	*β*	*p*
(Constant)		**1.8.E−39**	(Constant)		**6.7.E−26**
HAMD‐21 core	0.39	**1.7.E−05**	Age of onset	−0.21	**2.2.E−04**
Age	−0.28	**5.6.E−07**	Education	0.12	0.033
PSQI sleep disturbance	0.13	0.017	PSQI sleep disturbance	0.11	0.042
Duration of illness	0.11	0.037			
HAMD‐21 activity	−0.18	0.039			

3. Separation anxiety			4. Timidity		
Adjusted *R* ^2^ = 0.277	*β*	*p*	Adjusted *R* ^2^ = 0.089	*β*	*p*
(Constant)		**2.2.E−39**	(Constant)		**7.2.E−44**
HAMD‐21 core	0.44	**9.1.E−07**	HAMD‐21 core	0.14	0.024
PSQI total	0.19	**6.4.E−04**	Age	−0.17	**2.2.E−03**
Activity	−0.27	**1.5.E−03**	PSQI total	0.14	0.017
Number of lifetime suicide attempts	0.10	0.06			
Age	−0.28	**8.6.E−08**			
Duration of illness	0.19	**3.2.E−04**			
Psychotropic medication use	−0.11	0.034			

5. Fragile inner self			6. Total		
Adjusted *R* ^2^ = 0.218	*β*	*p*	Adjusted *R* ^2^ = 0.253	*β*	*p*
(Constant)		**3.9.E−10**	(Constant)		**7.3.E−66**
HAMD‐21 core	0.24	**7.2.E−04**	HAMD‐21 core	0.44	**6.4.E−07**
Age of onset	−0.22	**3.8.E−05**	Age	−0.32	**3.6.E−09**
HAMD‐21 delusion	0.20	**3.6.E−03**	Duration of illness	0.16	**1.4.E−03**
Body mass index	0.12	0.025	PSQI sleep disturbance	0.15	**2.8.E−03**
			HAMD‐21 activity	−0.24	**5.5.E−03**

Abbreviations: HAMD‐21, 21‐item Hamilton Depression Rating Scale; PSQI, Pittsburgh Sleep Quality Index.

Corrected significant *p*‐values are shown in bold exponents (*p* < 0.0083).

**Table 7:** Multiple regression analyses for the Interpersonal Sensitivity Measure scores in healthy individuals

1. Interpersonal awareness			2. Need for approval		
Adjusted *R* ^2^ = 0.226	*β*	*p*	Adjusted *R* ^2^ = 0.118	*β*	*p*
(Constant)		**2.7.E−87**	(Constant)		**2.0.E−98**
Age	−0.32	**3.5.E−15**	Age	−0.24	**2.7.E−08**
PSQI total	0.22	**1.0.E−06**	PSQI daytime dysfunction	0.11	**2.4.E−02**
PSQI daytime dysfunction	0.14	**2.2.E−03**	Sex	0.15	**2.6.E−04**
			PSQI total	0.10	0.043
			Number of lifetime suicide attempts	−0.08	0.047

3. Separation anxiety			4. Timidity		
Adjusted *R* ^2^ = 0.210	*β*	*p*	Adjusted *R* ^2^ = 0.102	*β*	*p*
(Constant)		**1.6.E−82**	(Constant)		**5.6.E−107**
Age	−0.29	**5.7.E−13**	PSQI daytime dysfunction	0.21	**1.6.E−06**
PSQI total	0.22	**1.1.E−06**	PSQI sleep latency	0.14	**9.5.E−04**
PSQI daytime dysfunction	0.14	**2.2.E−03**	Age	−0.13	**3.0.E−03**

5. Fragile inner self			6. Total		
Adjusted *R* ^2^ = 0.204	*β*	*p*	Adjusted *R* ^2^ = 0.245	*β*	*p*
(Constant)		**9.2.E−71**	(Constant)		**7.5.E−132**
Age	−0.32	**7.6.E−15**	Age	−0.32	**2.7.E−15**
PSQI total	0.17	**2.6.E−04**	PSQI total	0.22	**1.2.E−06**
PSQI daytime dysfunction	0.15	**9.4.E−04**	PSQI daytime dysfunction	0.17	**2.6.E−04**

*Note*: Sex was coded as male: 1 and female: 2.

Abbreviation: PSQI, Pittsburgh Sleep Quality Index.

Corrected significant *p*‐values are shown in bold exponents (*p* < 0.0083).

The correlations of the IPSM scores with continuous and ordinal variables in patients with schizophrenia, those with BD Ⅰ, those with BD Ⅱ, those with MDD, and healthy individuals are shown in Supporting Information: Tables S1–S5, respectively. Comparisons of the IPSM scores divided by dichotomous variables are shown in Supporting Information: Table S6. The interpersonal awareness score was significantly higher in female patients than in male patients with BD Ⅱ, whereas the need for approval score was significantly higher in female patients than in male patients with MDD (corrected *p* < 0.05). No significant difference was noted between the psychotropic medication use and drug‐free subgroups in any patient groups. Comparisons of the IPSM scores between lifetime suicide attempters and non‐attempters according to the number of attempts are shown in Supporting Information: Table S7. In patients with MDD, the separation anxiety score was significantly higher in one or more attempters, two or more attempters, three or more attempters, and four or more suicide attempters than in non‐attempters (corrected *p* < 0.05).

## DISCUSSION

The IPSM interpersonal awareness, separation anxiety, timidity, fragile inner self, and total scores were higher in all patient groups than in healthy individuals. Our findings on relatively long duration of schizophrenia (duration of illness: 14.1 ± 9.5 years) are consistent with those on at‐risk mental state (*n* = 62)^8^ and first‐episode psychosis (*n* = 34).^9^ Additionally, the IPSM separation anxiety, fragile inner‐self, and total scores were higher in patients with BD Ⅱ than in those with MDD, which is in line with the findings of a previous study (BD Ⅱ: *n* = 158; unipolar depression: *n* = 258).^11^ In addition, the IPSM scores were higher in patients with MDD than in healthy individuals, which is in line with the findings of a study on a history of depression (*n* = 306).^17^ As hypothesized, higher IPS was observed in major psychiatric disorders in a non‐specific manner. Our data may support that higher IPS plays a role of vulnerability factor associated with the development of major psychiatric disorders.

The PSQI total score and PANSS general psychopathology score were positively correlated with the IPSM total score in patients with schizophrenia. Among the symptoms of schizophrenia, sleep state and general psychopathology symptoms were well correlated with the IPSM scores (e.g., interpersonal awareness, separation anxiety, and fragile inner self) in our correlational analyses, suggesting that IPS in schizophrenia is principally based on the symptoms of disturbed sleep state and increased psychopathology score. These findings may support reviews on IPS in the psychopathology of patients with schizophrenia.^6^, ^7^

The HAMD‐21 delusion subscale score was positively correlated in patients with BD Ⅰ, whereas the HAMD‐21 total score was positively correlated in patients with BD Ⅱ, with the IPSM total score. These findings suggest that IPS is principally based on depressive symptoms, rather than manic symptoms, in patients with BD. Among depressive symptoms, delusion is well correlated with the IPSM scores, which may be reasonable because delusion symptoms defined in a previous study^32^ consisted of guilt, hypochondriasis, and paranoid symptom elements in the HAMD‐21.^35^ Moreover, according to our knowledge, we found for the first time that the IPSM need for approval score was higher in patients with BD Ⅰ and those with BD Ⅱ than in patients with other major psychiatric disorders (i.e., schizophrenia and MDD). This suggests that even higher need for approval among major psychiatric disorders may be especially related with a risk factor for the development of BD.

The HAMD‐21 core subscale score, duration of illness, and PSQI sleep disturbance subscale score were positively correlated, whereas the HAMD‐21 activity subscale score was negatively correlated with the IPSM total score in patients with MDD. As expected from previous studies that reported a positive correlation between the IPSM and depressive symptom scores (*n* = 88 and *n* = 306),^17^, ^19^ this study found that IPS was correlated with depression (especially its core symptoms) and that IPSM scores were positively correlated with sleep state in patients with MDD. In addition, the association of duration of illness with the IPSM scores suggested that IPS is a contributing factor for chronicity of MDD, which is in line with the associations of poorer outcome of depressive episode (*n* = 111),^16^ treatment‐resistant depression (*n* = 199),^18^ and delayed “return to work” (*n* = 306)^19^ with higher IPSM scores in patients with MDD. The negative association of the HAMD‐21 activity subscale score with work and interests and retardation elements^35^ suggested that these symptoms might diminish the interest in interpersonal relations in patients with MDD.

The PSQI total score and daytime dysfunction subscale score were positively correlated with the IPSM total score in healthy individuals. Our findings suggest that sleep state is closely related to IPS not only in patients with major psychiatric disorders (i.e., schizophrenia and MDD), but also in healthy individuals. Inconsistent with a prior study (*n* = 335),^20^ we found no sex difference between male and female sexes in healthy individuals. Furthermore, inconsistent with Joiner's Interpersonal Theory of Suicide^36^ and relevant prior studies,^37^, ^38^ lifetime suicide attempts were not associated with IPSM scores in patients with major psychiatric disorders, which may not be comparable with a previous study on the association between IPS and suicidal ideation in individuals without psychiatric disorders (*n* = 312).^21^ Unlike to our prior study on the Schizotypal Personality Questionnaire in patients with schizophrenia,^39^ the IPSM may not be a suitable assessment tool to predict suicidality in patients with major psychiatric disorders.

This study has several limitations. First, 83.7% of the patients with schizophrenia, 48.9% of those with BD Ⅰ, 60.9% of those with BD Ⅱ, and 50.2% of those with MDD had taken any psychotropic medication, although the effects were not significant on the IPSM scores. Second, the number of participants with schizophrenia (*n* = 166), those with BD Ⅰ (*n* = 47), and those with BD Ⅱ (*n* = 110) was relatively less than those compared with MDD (*n* = 380) or healthy individuals (*n* = 558), which may lead to lower statistical power in the schizophrenia, BD Ⅰ, and BD Ⅱ groups. Third, we could not distinguish early‐ and chronic‐stage psychosis in patients with schizophrenia as well as melancholic and non‐melancholic depression in patients with MDD since such detailed clinical information was not included in the present cohort. Therefore, patients with both types of psychosis and those with both types of depression were included in the schizophrenia and MDD groups, respectively. This study could for the first time reveal higher IPS in relatively long duration of patients with schizophrenia, whereas previous findings of higher IPS in non‐melancholic depression or no remarkable IPS alteration in melancholic depression^13^, ^14^, ^15^ could not be further supported. Fourth, statistical analyses revealed that IPSM scores were higher in patients with major psychiatric disorders and correlated with symptom scores; however, the IPSM scores were widely overlapped with those in healthy individuals and were not strong predictors for psychiatric symptoms. Differing from the hypothesis we expected, this study may rather support that IPS is relatively high in major psychiatric disorders and is related to some symptoms scores measured by different scales. Finally, the cross‐sectional design of this study could not address whether higher IPSM scores were the cause or result of major psychiatric disorders, or were associated with the psychopathology in the patients. Although we think that IPS is a relatively stable personality trait which acts as a non‐specific risk marker for psychiatric disorders, it may tend to fluctuate according to clinical state. In other words, the possibility of cause and result may be mixed, resulting in the formation of a vicious circle and their associations with clinical symptoms.

In conclusion, the IPSM scores were higher in all patient groups than in healthy individuals. The IPSM need for approval score was especially higher in patients with BD Ⅰ and those with BD Ⅱ than in the other diagnostic groups. The PSQI total score and PANSS general psychopathology score, HAMD‐21 delusion subscale score, HAMD‐21 total score, HAMD‐21 core subscale score, and PSQI sleep disturbance subscale score were positively associated with the IPSM total score in patients with schizophrenia, those with BD Ⅰ, those with BD Ⅱ, and those with MDD, respectively, while the PSQI total score and daytime dysfunction subscale score were positively associated with the IPSM total score in healthy individuals. These findings suggest that higher IPS may be associated with the development of major psychiatric disorders and some clinical symptom formations.

## AUTHOR CONTRIBUTIONS

Shinsuke Hidese designed and Hiroshi Kunugi supervised the study. Shinsuke Hidese and Miho Ota made diagnoses and evaluated the symptoms. Junko Matsuo, Ikki Ishida, Yuuki Yokota, and Kotaro Hattori recruited and interviewed the participants. Shinsuke Hidese performed the statistical analyses and wrote the manuscript, which was approved by all authors.

## CONFLICT OF INTEREST

The authors declare no conflict of interest.

## ETHICS APPROVAL STATEMENT

The study was approved by the ethics committee of the NCNP.

## PATIENT CONSENT STATEMENT

Written informed consent form was gained from all participants.

## Supporting information

Supporting information.

## Data Availability

No data sharing.
